# Elevated plasma interleukin-37 playing an important role in acute coronary syndrome through suppression of ROCK activation

**DOI:** 10.18632/oncotarget.14195

**Published:** 2016-12-25

**Authors:** Tengyu Yang, Fang Fang, Yawen Chen, Jing Ma, Zhaowen Xiao, Songfeng Zou, Na Zheng, Dewen Yan, Songyan Liao, Shaoyuan Chen, Hongchen Fang, Chekmen Yu, Jie Liu, Ming Dong

**Affiliations:** ^1^ Division of Pathophysiology, Medical College, Shenzhen University, Shenzhen, Guangdong, China; ^2^ Division of cardiology, Department of Medicine and Therapeutics, Prince of Wales Hospital, Li Ka Shing Institute of Health and Sciences, Institute of Vascular Medicine, The Chinese University of Hong Kong, Hong Kong, China; ^3^ Cardiology Division, Department of Medicine, The Nanshan Hostipal, Shenzhen, Guangdong, China; ^4^ Department of Endocrinology, The First Affiliated Hospital of Shenzhen University, Shenzhen, China

**Keywords:** interleukin -37, acute coronary syndrome, ROCK activity

## Abstract

**Objective:**

The plasma level of interleukin-37 is elevated in patients with acute coronary syndrome, however, its function during the onset and progress of the disease remains unclear. This study aimed to investigate the clinical significance of IL-37 in acute coronary syndrome and its underlying mechanism.

**Methods:**

124 patients with acute coronary syndrome and 40 healthy controls were recruited in this study. Plasma interleukin-37 levels were measured in 41 patients with ST elevation myocardial infarction (STEMI), 41 patients with non-STEMI, 42 patients with unstable angina, and 40 controls. Mortality was defined as an event.

**Results:**

In this study, the mean follow-up period was 824±306 days (2-1077 days). 22% (n=27) of patients died. The mortality rate was significantly lower in patients with interleukin-37 serum levels below the median (6.4 pg/mL) than those with interleukin-37 serum levels above 6.4 pg/mL at 36-month follow-up (16% vs. 24%, p=0.02, log rank X^2^=5.39). Highly concentration of the anti-inflammatory interleukin-37 exerted a protective effect by suppressing the activated Rho Kinase (ROCK) activity in the peripheral blood mononuclear cells *in vivo and in vitro* after ischemia/reperfusion injury and stimulation of the Rho activator, calpeptin.

**Conclusions:**

The interleukin-37 level is significantly increased in acute coronary syndrome. Elevated baseline interleukin-37 levels in patients on admission are associated with poor outcomes. Thus, we propose that interleukin-37 could be a biomarker predictive of mortality in acute coronary syndrome. Moreover, this study reveals that the protective effect of interleukin-37 against atherosclerosis may involve the inhibition of ROCK activity.

## INTRODUCTION

Acute coronary syndrome (ACS) is the most common cause of morbidity and death globally. The imbalance between pro-inflammatory cytokines and anti-inflammatory cytokines is associated with progression of atherosclerosis, plague instability, and symptoms in patients [1, 2].

Interleukin-37 (IL-37) is a newly defined member of the interleukin-1 (IL-1) family, and a pivotal anti-inflammatory cytokine in the inflammatory regulation [3]. In healthy individuals, due to the possible instable elements within the coding region, steady-state IL-37 mRNA and protein are expressed in low levels in monocytes, dendritic cells, and plasma cells [6, 7]. The transgenic expression of human IL-37 in a mouse macrophage line suppresses significantly the production of pro-inflammatory cytokines and chemokines [3]. Plasma IL-37 has been shown to increase in ACS [8]. We previously reported a possible strong association between IL-37 and atherosclerosis as discovering IL-37 in the foam-like cells of carotid artery plagues in rabbits [9].

Rho-kinase (ROCK) activity is increased in atherosclerosis and acts as a marker of atherosclerotic burden. ROCKs involve in the regulation of vascular tone, endothelial dysfunction, inflammation and remodeling, and are regulated by an intricate network of signaling cascade. According to our previous finding, ROCK activity is increased in mouse myocardiocytes after ischemia/reperfusion injury (I/R) (data on submitting) and is also increased in peripheral leukocytes of patients with ACS [10]. ROCKs involve in regulations of various pro-inflammatory cytokines (such as NF-kB, PKC, IL-6) that participate in the process of atherosclerosis [11–13]. In this study, we evaluated the function of IL-37 in ACS patients at baseline, and investigated on the association between IL-37 and the risk of complicated cardiovascular events in these patients. Furthermore, we studied the correlation between IL-37 and ROCK activity and the effect of IL-37 on ROCK activity.

## RESULTS

### Baseline characteristics

Baseline characteristics and medications were recorded, including age, gender, blood pressure, smoking status, etc (Table 1). There was no significant difference in age, gender, blood pressure, heart rate or smoking status among STEMI, NSTEMI and UA groups. Of these three groups, the number of white blood cells was increased remarkably in the STEMI group. The level of fasting glucose was significantly higher in STEMI group than that in UA group. The peak concentrations of cTnT and CPK were significantly higher in STEMI group than those in both UA and NSTEMI groups. Other parameters are described in Table 1.

**Table 1:** Baseline Characteristics of acute coronary syndrome and control subjects

Clinical variables	NCn=40	UAn=42	NSTEMIn=41	STEMIn=41
Age (yrs)	67.29±9.58	64.00±12.54	70.31±14.90	66.94±16.35
Gender (male)	26(65%)	29(69%)	25(61%)	30(73%)
Current smoker	0	21(50%)	16(39%)	20(49%)
SBP (mmHg)	129.24±22.24	152.30±28.92	154.22±26.21	135.0±031.42
DBP (mmHg)	77.96±13.20	82.93±14.44	84.72±16.24	72.31±18.66
HR (/minute)	82.62±24.75	81.30±15.27	87.53±20.09	82.62±24.75
LVEF	75.12±8.41	57.12±11.53	45.32±11.54*	35.42±7.35*
BMI (kg/m^2^)	19.21±2.14	20.14±2.63	21.78±3.54	22.41±3.87§
WBC (x10^9^/l)	5.65±1.38	7.91±2.66	9.65±3.77	12.70±3.44*
Creatinine (μmol/l)	71.93±19.12	86.93±16.59	150.50±187.00	155.81±160.86
Fasting glucose (hightest) (mmol/l)	5.21±0.33	5.80±1.18	6.65±2.49	7.18±2.30
HbA1c (mmol/mol)	47.62±14.65	47.05±9.08	52.25±13.05	50.00±13.87
TG (mmol/l)	1.36±0.63	1.58±0.92	1.71±0.88	1.79±1.50
TC (mmol/l)	5.16±0.54	4.86±1.30	4.54±0.82	4.71±0.82
HDL-C (mmol/l)	1.20±0.39	1.15±0.28	1.10±0.33	1.02±0.34
LDL-C (mmol/l)	3.02±0.37	3.07±1.18	2.67±0.71	2.96±0.84
Peak cTnT (μg/l)	NA	0.023±0.03	0.76±1.12	3.80±5.77*
Peak CPK (U/L)	NA	255.80±371.35	641.46±834.57§	2667.31±1974.44*
Treatment				
PCI	NA	18(42%)	22(54%)	24(59%)
CABG	NA	0	0	4(10%)*
Aspirin	NA	39(93%)	39(95%)	40(98%)
Clopidogrel	NA	12(28%)	19(46%)	31(76%)
LMWH	NA	35(83%)	37(90%)	36(87%)
Statin	NA	27(64%)	32(78%)	39(95%)

* p<0.05 vs NA, UA, NSTEMI, # p<0.05 vs NSTEMI, §p<0.05 vs NA and UA

The data are given as the mean ± SE or number of patients. SBP=systolic blood pressure, DBP=diastolic blood pressure, HR=heart rate, BMI=body mass index, WBC=white blood cell, HbA1c=glycoseylated hemoglobin, TG=Triglyceride, TC=Serum total cholesterol, HDL-C=High density lipoprotein cholesterol, LDL-C=Low density lipoprotein cholesterol, Peak cTnT=Peak troponin T, Peak CPK=Peak creatine kinase; PCI= Percutaneous Coronary Intervention; CABG= coronary artery bypass graft; LMWH= low molecular weight heparin

### Expression levels of plasma IL-37 in normal controls and ACS subgroups

**Figure 1:**
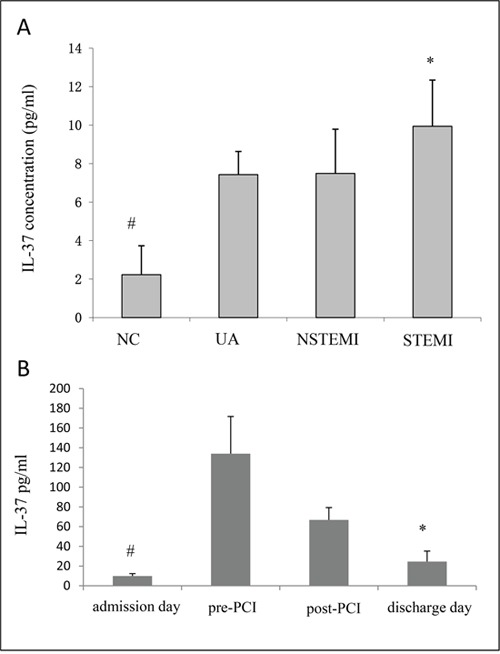
**A.** The expression of plasma IL-37 was markedly higher in the ACS than that in the controls (NC), and the expression of IL-37 in STEMI was especially highest among all the subgroups. # p<0.05 vs UA, NSTEMI and STEMI, *p<0.05 vs UA and NSTEMI. **B**. The expression of plasma IL-37 level in severe ACS (STEMI) patients at different time points. IL-37 was dramatically increased before PCI and decreased before discharge. However, it was still higher than that at admission. # p<0.05 vs pre-PCI, post-PCI and discharge day, *p<0.05 vs pre-PCI and post-PCI.

Values of IL-37 expression level varied widely from 2.9 pg/ml to 783 pg/ml among individuals. The IL-37 expression level was higher in ACS patients (8.76±3.32 pg/ml) than normal controls (2.23±1.54 pg/ml) (p<0.001). Of three ACS subgroups, the plasma IL-37 concentration was significantly higher in STEMI group (9.94±2.42 pg/ml) than both UA (7.43±2.31 pg/ml) and NSTEMI (7.49±1.20 pg/ml) groups (all p<0.001). Nevertheless, there was no significant difference in the serum IL-37 level between UA and NSTEMI groups (both p<0.05) (Figure 1A).

Moreover, periphery blood was collected in the STIMI group on admission, pre-PCI, post-PCI and discharge days for investigation of IL-37 expression in acute disease conditions. The IL-37 concentration was increased steadily before PCI (admission: 9.94±2.42 pg/ml, pre-PCI: 133.93±37.75 pg/ml, post-PCI: 66.85±12.47 pg/ml, discharge: 24.60±10.56 pg/ml) (Figure 1B). Even though IL-37 concentration was decreased after PCI treatment, it remained above the baseline level.

### IL-37 as a predictor of mortality for ACS patients

**Figure 2:**
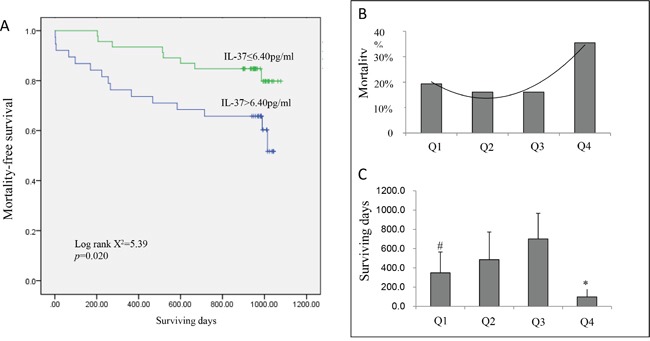
**A.** The Kaplan–Meier curves for death-free survival within 3 years according to median of IL-37 (6.4 pg/ml). High IL-37 group has higher mortality risk to low IL-37 expression group (log rank X^2^ = 5.16, p = 0.023). **B**. Mortality and surviving days **C**. in ACS patients according to quartile of plasma IL-37 level. Q4 had a significantly higher mortality than Q1, Q2 and Q3 (p<0.05). However, Q1 had relative higher mortality and shorter surviving days than Q2 and Q3 (p<0.05). # p<0.05 vs Q2 and Q3, *p<0.05 vs Q1, Q2 and Q3.

In order to examine whether plasma IL-37 could be used to predict long-term mortality, patients were followed up for a mean period of 824±306 days (range, 2 to 1077 days). One patient failed to be recorded, and 27 patients (22%) died. The possible explanation for relatively high mortality might be the high average age (≈70 years) of patients. The baseline serum level of IL-37 was found to relate to the long-term prognosis for ACS patients (Table 2). The mortality rate was significantly lower in patients with IL-37 serum levels below the median level (6.4 pg/ml) than above the median level (6.4 pg/ml) during 36-month follow-up (16% vs. 24%, p=0.02, log rank X^2^=5.39; Figure 2A).

**Table 2:** Multiple regression analysis of clinical variables and outcomes

Variables	Multivariate (enter)		P value
	HR	95%CI	
Gender	1.023	0.312-3.348	0.971
Age	1.054	1.004-1.106	0.033*
ACS group	1.718	0.027-0.889	0.037*
Smoker	0.842	0.894-1.060	0.530
PCI	1.039	0.226-3.130	0.797
IL-37	1.002	1.001-1.004	0.043*

HR, hazard ratio; PCI, percutaneous coronary intervention, ACS group, UA and NSTEMI vs STEMI.

* p<0.05

Based on the percentile of plasma IL-37 concentrations on admission, patients were divided into four groups: 2.97–4.54 ng/ml (Q1, first quartile), 4.64–6.13 ng/ml (Q2, second quartile), 6.41–10.2 ng/ml (Q3, third quartile), and 10.45–783.52 ng/ml (Q4, fourth quartile). Of the four quartiles, Q4 had a remarkably high mortality rate (p<0.05). Q1 had a relatively higher mortality rate and a shorter life expectancy than Q2 and Q3 did (p<0.05) (Figure 2B–2C).

### Plasma IL-37 expressions of different white blood cell (WBC) quarters in ACS patients

**Figure 3:**
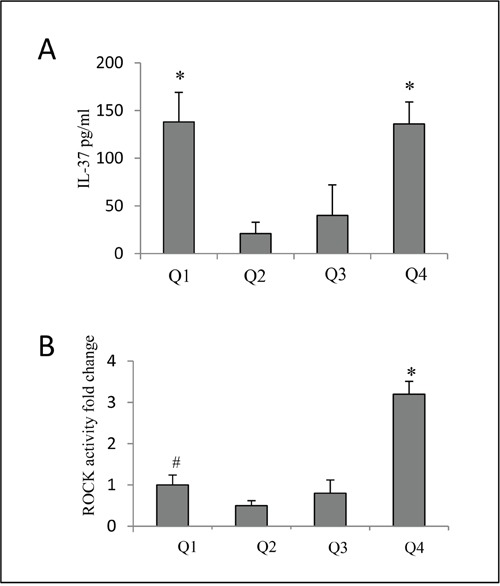
**A.** Expression of plasma IL-37 of different WBC quarters in ACS patients. Q1 and Q4 had significantly higher IL-37 than that in both Q2 and Q3 (p<0.01). *p<0.05 vs Q2 and Q3. **B**. Thus we observed ROCK activity in different baseline IL-37 quartiles. ROCK activity was significantly increased in Q4 of IL-37 than Q1, Q2 and Q3. The ROCK activity in Q1 was also increased than Q2. # p<0.05 vs Q2, *p<0.05 vs Q1, Q2 and Q3.

As PBMCs is the main constituent of white blood cells (WBCs), the IL-37 levels of different WBC quarters were compared using ANOVA analysis. The IL-37 levels of Q1 and Q4 were 138±31 pg/ml and 136±23 pg/ml, respectively. The IL-37 levels of Q2 and Q3 were 21±12 pg/ml and 40±32 pg/ml, respectively. The IL-37 levels of Q1 and Q4 were remarkably higher than that of both Q2 and Q3 (all p<0.01) (Figure 3A).

Previous evidence proved that inflammatory reaction was associated with ROCK activity in ACS [10]. Thus we observed ROCK activity in different baseline IL-37 quartiles. ROCK activity was significantly increased in Q4 of IL-37 than Q1, Q2 and Q3.(p<0.05). The ROCK activity in Q1 was also increased than Q2. (p<0.01) (Figure 3B).

### Expression of plasma IL-37 and ROCK activity of PBMCs in STEMI patients on admission and at discharge

**Figure 4:**
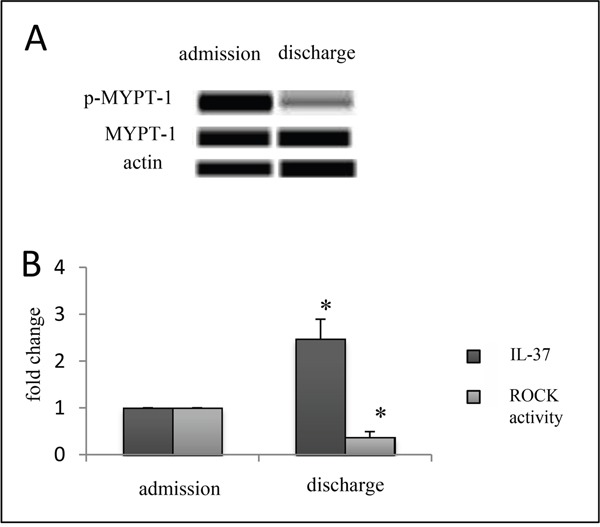
Expression of plasma IL-37 and ROCK activity of PMBCs in patients with STEMI on both admission and discharge times Western blotting results showed the decreased ROCK activity of PBMCs **A**. but still higher plasma level of IL-37 on discharge **B**. n=6, Error bars are shown as mean±s.e.m. *p<0.05 vs admission (one-way analysis of variance).

ROCK activity, which induces inflammation, is higher in ACS patients than normal controls [10]. We hypothesized that IL-37 might be positively correlated with ROCK activity at baseline due to its possible inhibition on the ROCK activation and the homeostasis between IL-37 and ROCKs. Since STEMI is a severe inflammatory condition, we investigated the correlation between plasma IL-37 and ROCK activity in PBMCs under STEMI condition. Although plasma IL-37 was increased before PCI and decreased continuously till discharge, it remained above the baseline level (around 2.5 times) (Figure 4B). However, the ROCK activity in PBMCs was originally increased, but eventually declined to 37% lower than that on admission (Figure 4A, 4B).

### Suppression of increased ROCK activity in healthy donor PBMCs after ischemia reperfusion (I/R) via IL-37 protein

**Figure 5:**
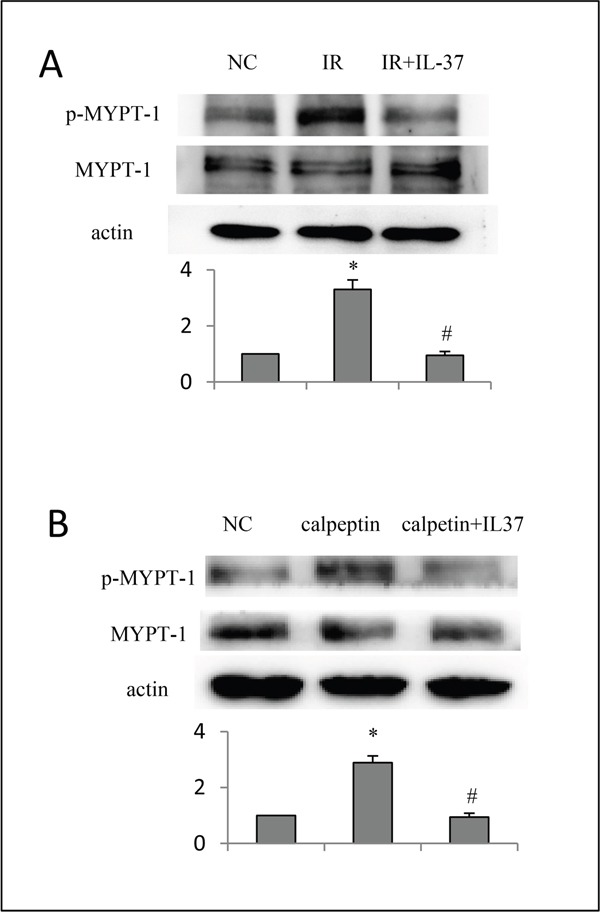
**A.** Protection of IL-37 in healthy donor cells (PMBCs) *in vitro* with ischemial reperfusion injury. **B**. IL-37 decreased ROCK activity in healthy donor cells (PMBCs) *in vitro* when stimulated by calpeptin.n=6, Error bars are shown as mean±s.e.m. *p<0.05 vs normal control, # p<0.05 vs IR or calpeptin group (one-way analysis of variance).

We observed a possibly positive correlation between IL-37 and ROCK activity in healthy donor PBMCs *in vitro*. Firstly, we observed IL-37 expression in human PBMCs *in vitro*. 100 ng/ml lipopolysaccharide was added to PBMCs to stimulate inflammation. Data showed that IL-37 increases to 12 pg/ml in the cell supernatant. However, the concentration of IL-37 in control was too low to detect. Secondly, the phosphorylation level of MYPT-1 was remarkably higher in I/R group than normal controls. ROCKs were activated after I/R. An obvious down regulation of phosphorylated MYPT1 (p-MYPT1) in I/R + IL-37 group was observed compared to I/R group (Figure 5A). Treatment with IL-37 reduced the ROCK activity. The inhibitory effect of IL-37 on ROCKs was further confirmed by using ROCK activator, calpeptin. The cultures that were treated with IL-37 for 24 hours were incubated for 30 minutes with calpeptin (Figure 5B). IL-37 reversed the calpeptin-induced ROCK activity and caused a complete decline of ROCK activity. This observation further confirmed the inhibitory effect of IL-37 on ROCK activation.

## DISCUSSION

The major findings of this study are as following: the serum level of anti-inflammatory cytokine IL-37 is higher in patients with ACS than healthy controls; elevated baseline IL-37 concentration on admission is associated with a poor outcome; the high concentrated IL-37 suppresses the increased ROCK activity in PBMCs after IR injury or stimulation of calpeptin *in vitro*. These results further support that the balance between pro-inflammatory and anti-inflammatory cytokines determines the outcome of ACS patients.

We previously reported that the plasma level and expression of IL-37 [5, 17] of the plaque are significantly increased in the pathogenesis of atherosclerosis, a chronic inflammatory process [9]. In this study, high concentrations of IL-37 in ACS patients were observed on admission, consistent with the findings by Ji, *et al*. [8]. The measured concentrations of IL-37 varied widely in ACS patients, but were too low in healthy controls to be detected by ELISA. It has been reported that the plasma level of IL-37 in ACS patients is positively correlated with the levels of inflammatory biomarkers C-reactive protein (CRP) and interleukin-18 (IL-18) [8]. Moreover, IL-37 exerts an anti- inflammatory effect by attenuating the production of inflammatory cytokines, such as TLR, IL-18, TNFα, etc. [18]. Thus, the rising plasma IL-37 level in ACS patients may result from inflammatory activation. The higher IL-37 level reflects a greater inflammatory response. Thus, the elevated IL-37 level in ACS patients may be a compensatory response to excessive inflammation. Different anti-inflammatory reactions in patients with acute-stress status might be a major cause of various measured IL-37 values.

The high baseline IL-37 expression was observed to result in a higher mortality than the low baseline IL-37 expression did. IL-37 might be predictive of the long-term outcome. Increased pro-inflammatory activity promotes the synchronous increasing in anti-inflammatory activity. This protective mechanism might explain the positive correlation between expression of IL-37 during ACS and the disease severity. IL-37 has been considered as a protective element in atherosclerosis. However, the underlying mechanism, by which IL-37 attenuates the symptom of atherosclerosis, remains unclear.

The plasma IL-37 level of ACS patients was found to be dramatically increased after admission and decreased before discharge. The major factors might be increasing inflammatory responses of patients in the beginning and the effects of anti-inflammatory and lipid-lowering treatments they received in hospital. The high IL-37 level indicates a strong immune reaction. On the contrary, the extremely low IL-37 level in acute disease states reflects a weak immune system. Therefore, the mortality rate of Q1 was higher than that of Q2 and Q3.

ROCK activity induces inflammation and is increased in ACS [10]. The activation of ROCK activity enhances expressions of pro-inflammatory cytokines, whereas the inhibition of ROCK reduces productions of those cytokines [19]. In this study, after anti-inflammatory and lipid-lowering treatments, we found that ROCK activities in patients were lower than that on admission. However, IL-37 levels remained higher than that on admission. This observation suggests that the immune reaction may take place later than the inflammatory reaction does. Furthermore, IL-37 might have a treatment function for ACS via inhibition of ROCK activation, according to our observation that IL-37 reversed the increased ROCK activity in cultured PMBCs after ischemia/reperfusion injury or the ROCK stimulus. The physiological concentration of IL-37 in human is around picogram per milliliter. However, the therapeutic dose *in vitro* is around nanogram per milliliter, which is around one thousand times. That is why IL-37 could exert a protective role through inhibition of ROCK activation *in vitro*. Rho-kinase pathway exerts deleterious effects on many diseases, such as diabetes, pulmonary hypertension, and cancer. To the best of my knowledge, it was the first time to elaborate the correlation of IL-37 and ROCK. However, further studies are required to examine whether IL-37 has the similar function in other ROCK-related diseases and whether such high concentration is safe in human. Previously, we discovered that the rosuvastatin treatment dramatically lowered the IL-37 level in rabbits with atherosclerosis, a chronic inflammatory process [9]. Thus, IL-37 could be a promising therapeutic medium to treat atherosclerosis.

We propose that the plasma IL-37 level could be predictive of mortality in ACS patients. Furthermore, we report for the first time that IL-37 may exert a cardio-protective effect by suppressing the ROCK activity. These findings provide new insights for the research of the function of IL-37, and suggest its possible role in the prognosis of ACS, and as a new therapeutic target of ACS. Further studies are required to reveal the mechanism, by which IL-37 may inhibit ROCK activation, and investigate the treatment function of exogenous IL-37 for ACS.

## MATERIALS AND METHODS

### Subjects

From January 2012 to December 2014, patients (n=124, 69%men, mean age: 68±15years) who were diagnosed with ACS and hospitalized at Prince of Wales hospital (Hong Kong) were recruited in this study. ACS was diagnosed following the ACC/AHA guideline [14]. Patients who entered the registry had to be at least 18 years old, alive at the time of hospital presentation, admitted for ACS as a presumptive diagnosis, and have at least one of the followings: electrocardiographic changes consistent with ACS, serial increases in serum biochemical markers of cardiac necrosis, and/or documentations of coronary artery diseases. Written informed consents were obtained from all subjects on admission. Patients with qualified ACS precipitated or accompanied by a significant comorbidity, trauma, or surgery were excluded from this study. Enrolled patients were divided into ST elevation myocardial infarction (STEMI), non-STEMI (NSTEMI) and unstable angina (UA) groups. All ACS subgroups were matched in age, gender, and smoking status. Patients were followed up for a mean period of 27.0±10.1 months (range, 1.2 to 35.4 months) or until death.

Healthy control group (n=40, 65% men; aged 67±9 years) was recruited depending on the presence of hypertension and smoking status, which influence ROCK activity. The inclusive criteria for normal controls were: no history of cardiovascular or systemic illness, results of regular physical examinations including blood pressure measurement, normal hemoglucostix and ECG, no echocardiographic evidence of structural or functional heart diseases and no ongoing medication. Written informed consents were obtained from these subjects. All control subjects underwent coronary angiography to ensure healthy epicardial coronary arteries.

### Blood samples

Blood samples were collected from subjects at four time points (within 24 hours after emergency admission, pre percutaneous coronary intervention (pre-PCI), post percutaneous coronary intervention (post-PCI), and discharge day). Blood samples were centrifuged at 2000×g for 20 min at room temperature. The serum and peripheral blood were collected and stored at −80°C for further use.

### ELISA

Plasma IL-37 (Rapidbio, USA, Catalogue number:IR299) values were measured by enzyme-linked immunosorbent assays (ELISA). The minimal detectable concentration of IL-37 was 1 pg/ml. All samples were handled in duplications and in accordance with the manufacturers’ protocols.

### PBMCs isolation and *in vitro* culture

Peripheral blood mononuclear cells (PBMCs) (monocytes and lymphocytes) were isolated from human buffy coats with the technique from Panda *et al*. [15]. To prepare an ischemic reperfusion disease model, PBMCs were then treated with 1% O_2_ for 2 hours and incubated on the non-glucose and serum-free DMEM culture media for 4 hours in normal condition for cell growth. PBMCs were treated with human IL-37 protein (Sino Biological Inc: 10155-HNAE, 25 ng/ml) before I/R injury. After IL-37 treatment, some of the cells were incubated with 10 uM Rho activator calpeptin (Santa Cruz Biotechnology) for 30 minutes [16].

### Western blot

The total protein was measured via a bicinchoninic acid assay kit (Pierce) after sonication. 15 ug per well protein was loaded on 7.5% polyacrylamide gels, transferred onto nitrocellulose paper, and stained with primary and secondary antibodies. Reactive bands were visualized using the SuperSignal ECL Western blotting detection kit (Pierce), and the densitometry was obtained via the ImageJ software. In this study, myosin phosphatase was inactivated by ROCK through the specific phosphorylation of myosin phosphatase target subunit 1 (MBS or MYPT1) at Thr-853. This resulted in an increase in the phosphorylated content of the 20-kDa myosin light chain. Therefore, the ratio of pMBS to MBS was used to represent the ROCK activity. The primary monoclonal antibodies used in the western blot were anti-MYPT-1, phosphor T853-MYPT1 and IL-37, which were all from Santa Cruz Biotechnology.

### Statistical analysis

All data were given as mean ± SD. Student's t-test was used to compare between every two groups. One-way ANOVA followed by the Neuman-Keuls post hoc test was used for comparisons among 3 or more groups. Spearman's correlation was used to calculate the correlations between the plasma biomarker level and other measured parameters. In all tests, a value (p < 0.05) was considered to be statistically significant.

## LIMITATIONS

The number of enrolled ACS patients in this study was small. Further study is needed with a larger number of subjects to confirm the significance of IL-37 in long-term mortality.
